# Correlation between CH_2_DS_2_-VASc Score and Serum Leptin Levels in Cardioembolic Stroke Patients: The Impact of Metabolic Syndrome

**DOI:** 10.1155/2017/7503763

**Published:** 2017-10-31

**Authors:** Anna Szczepańska-Szerej, Jacek Kurzepa, Aneta Grabarska, Joanna Bielewicz, Ewa Wlizło-Dyś, Konrad Rejdak

**Affiliations:** ^1^Department of Neurology, Medical University of Lublin, Lublin, Poland; ^2^Department of Medical Chemistry, Medical University of Lublin, Lublin, Poland; ^3^Department of Biochemistry and Molecular Biology, Medical University of Lublin, Lublin, Poland

## Abstract

**Objective:**

To determine adipokines levels in patients with different etiologic subtypes of acute ischemic stroke (AIS) and metabolic syndrome (MetS) status.

**Methods:**

Serum adiponectin, leptin, and resistin levels were determined by ELISA in 99 AIS patients and 59 stroke-free control group subjects. Stroke patients were grouped based on MetS, modified TOAST classification, and CHA_2_DS_2_-VASc scale in case of cardioembolic stroke following atrial fibrillation.

**Results:**

No differences were found in all adipokine serum levels between AIS patients and appropriately matched control group. MetS-AIS patients had significantly higher leptin levels (22.71 ± 19.01 ng/ml versus 8.95 ± 9.22 ng/ml, *p* < 0.001) and lower adiponectin levels (10.71 ± 8.59 ng/ml versus 14.93 ± 10.95 ng/ml, *p* < 0.05) than non-MetS-AIS patients. In patients with cardioembolic stroke, leptin levels were significantly higher than in remaining stroke cases (19.57 ± 20.53 ng/ml versus 13.17 ± 12.36 ng/ml, *p* < 0.05) and CHA_2_DS_2_-VASc score positively correlated with leptin levels only (*p* < 0.001). Analysis of individual components of CHA_2_DS_2_-VASc score showed that hypertension, female gender, and diabetes had greatest impact on elevated serum leptin level.

**Conclusion:**

This pilot study revealed that leptin could be a potential biomarker for risk stratification of cardioembolic stroke in MetS patients and that heterogeneity of stroke subtypes should be considered for more refined and precise clinical stroke studies.

## 1. Introduction

Abdominal fat tissue has been considered to be an energy storage for many years, but along with the discovery of adipokines, it has recently been highlighted as an active endocrine organ. This discovery has opened new fields of research. Adipose tissue produces several adipokines which modulate insulin sensitivity and appears to play an important role in the pathogenesis of diabetes, dyslipidemia, inflammation and, in consequence, also in the pathophysiology of cardiovascular diseases [1, 2]. Abdominal obesity described by waist circumference (WC) has been shown to be more strongly associated with the risk of cardiovascular events than body mass index (BMI) [3].

Stroke is a major cause of disability and death in developed countries. Its prevalence is expected to increase in the future because of population aging [4]. Ischemic stroke, like other cardiovascular diseases, is the result of a number of risk factors such as age, hypertension, diabetes mellitus, smoking, left ventricular hypertrophy, atrial fibrillation (AF), and others. They are generally divided into nonmodifiable and modifiable ones, the latter of which are more interesting to physicians due to a possibility of therapeutic intervention. One of the newly considered modified risk factors is central obesity and, related to it, a metabolic syndrome (MetS) which represents a “constellation” of lipid and nonlipid risk factors for cardiovascular diseases. The contribution of obesity to the pathophysiology of stroke is very complex and multifactorial. Obesity is a precursor and usually coexists with hypertension, diabetes, and proatherogenic dyslipidemia which play an important role in the epidemiology of ischemic stroke. Direct contribution to the pathophysiology of ischemic stroke via adipokines is also considered [5].

Among main adipokines, leptin, adiponectin, and resistin seem to be stable, in contrast to ghrelin, visfatin, and others which are labile and are dependent on different conditions. Leptin is an adipokine with multidirectional action, which informs the brain about body energy reserves, suppresses appetite, and modulates energy consumption [6]. It also affects metabolism of lipids, insulin, glucose, and triglycerides [5–8]. Several experimental studies have shown that an increased level of leptin has a proinflammatory potential and is associated with systemic atherosclerosis, coronary heart disease, and ischemic stroke [9–11]. It has been shown that its serum concentration is directly related to the content of adipose tissue [5], although leptin production has been also observed in the stomach, the skeletal muscles, the liver, and the placenta [12]. Resistin is implicated in atherosclerosis by exerting inflammatory effects on endothelial cells and by decreasing endothelial NO synthase and endothelin 1 release, which upsets the endothelial function [13]. It was also suggested that resistin might play an important role in the pathogenesis of insulin resistance [7]. However, data from clinical trials did not confirm findings from animal studies. Resistin is produced in the stromal vascular fraction of the adipose tissue and in peripheral blood monocytes, but there is no correlation between body weight, adiposity, or resistin mRNA concentration [14]. In contrast to the proatherogenic potentials of leptin and resistin, experimental studies have revealed that adiponectin is abundantly expressed in healthy individuals. It also has complex antithrombotic, antiatherogenic, and anti-inflammatory properties [15, 16]. Adiponectin is a protein synthesized only by adipocytes. Unlike other adipokines, serum concentration of adiponectin is inversely correlated with BMI and WC [6, 7, 17]. Lower plasma adiponectin levels have been shown to be associated with insulin resistance, type 2 diabetes, and proatherogenic dyslipidemia as well as with an increase in the level of proinflammatory markers and endothelium disorders. They are also connected with essential hypertension, development of inflammation, atherosclerosis, and, in consequence, with cardiovascular diseases. From the prognostic viewpoint, in healthy individuals, adiponectin has been inversely associated with cardiovascular risk or directly associated with life expectancy [18, 19].

A number of studies have been published on the relation of adipokines to cardiovascular risk and the possibility of using them as prevention markers [20–22]. However, ischemic stroke is a heterogenic disease and the association between its etiologic subtypes and adipokines has not yet been established. In this context, our research hypothesis was that the strength of association of adipokine levels with ischemic stroke would differ between its particular etiologic subtypes. Additionally, our objective was to determine adipokine levels in acute ischemic stroke (AIS) patients according to MetS status.

## 2. Methods

### 2.1. Participants and Study Design

This was a single-center hospital-based prospective study. The authors collected consecutive AIS patients who had been admitted to the Stroke Unit/Neurological Department of the Medical University of Lublin from September 2012 to December 2013. Ischemic stroke was defined as a sudden focal neurologic defect lasting for more than 24 h and diagnosed on the basis of clinical history, neurological examination, and brain imaging by computed tomography (CT) or magnetic resonance imaging (MRI). Exclusion criteria included time from stroke onset and hospitalization > 24 h, infection on admission, autoimmune or malignant neoplasm diseases, previous surgical procedure, or extensive injury. The remaining 99 patients with AIS were included in the study. The study was approved by the Ethics Committee of the Medical University of Lublin (approval number-0254/126/2009), and the participating subjects or their legal representatives signed an informed consent.

We also recruited 59 control patients with negative medical history of stroke or transient ischemic attack (TIA), who voluntarily agreed to participate in the study. The chief reasons for their hospitalization were dizziness without central nervous system pathologies, mild cognitive impairment, low back pain, early Parkinsonism, essential tremor, and clinical evaluation without acute illness. Exclusion criteria for controls were the same as for AIS cases, and both groups were balanced according to age, sex, and MetS presence.

### 2.2. Clinical Measurements

Data on demographics, past medical history, and conventional stroke risk factors were collected for both patients and controls and included the following factors: gender, age, height, weight, waist circumference, hypertension, diabetes, MetS presence, AF, coronary heart disease (CHD), congestive heart failure, past stroke or hemispheric TIA, hypercholesterolemia, and peripheral arterial disease as well as current and past smoker and current drinker. Among current medications, treatment with angiotensin-converting enzyme inhibitors (ACE-I), angiotensin receptor-II inhibitors (AT-II), and statins was used in the study analyses. BMI was calculated as weight (in kilograms) divided by height (in meters) squared. MetS was diagnosed using the uniform criteria elaborated by the International Diabetes Federation (IDF), American Heart Association (AHA), National Heart, Lung, and Blood Institute (NHLBI), World Heart Federation (WHF), International Atherosclerosis Society (IAS), and International Association for the Study of Obesity (IASO) and published in 2009 [23]. Patients having three or more of the following abnormalities were defined as having MetS:

1. Abdominal obesity: waist circumference > 102 cm in men or >88 cm in women
2. Hypertension (prior diagnosis of hypertension, current blood pressure-lowering medication, or a systolic/diastolic blood pressure of 130/85 after neurological and medical stabilization)
3. Hypertriglyceridemia: serum triglycerides ≥ 150 mg/dL (1.7 mmol/l)
4. Low HDL-cholesterol: serum HDL-cholesterol < 40 mg/dL (0.9 mmol/l) in men or <50 mg (1.3 mmol/l) in women
5. Diabetes (prior diagnosis of diabetes, current glucose-lowering medication, or a fasting glucose level ≥ 100 mg/dl after neurological and medical stabilization)

For all patients, subtypes of IS were determined according to modified TOAST stroke type classification system [24]. Six different stroke subtypes including large artery atherosclerosis (LAA), cardioembolic (CE), small vessel occlusion (SVO), other determined etiology, undetermined etiology, and unclassified etiology were defined. All patients were categorized into one of these subtypes. Modification depended on introducing unclassified etiology in cases when more than one probable reason for ischemic stroke has been found. In further clinical evaluation, strokes of other determined etiology, undetermined etiology, and unclassified etiology were included in one group named “other etiology.” In the following division, all AIS patients were categorized into cardioembolic strokes and noncardioembolic strokes. The latter were composed of all the remaining types of etiology. For more precise discrimination between all stroke subtypes, extensive diagnostic procedures were performed. All AIS patients underwent carotid duplex and transcranial color-coded Doppler (TCCD) examinations for the detection of any stenotic extracranial or intracranial vascular lesions and angio-CT/angio-MRI in selected cases. Other studies included Holter-ECG and transthoracic or transesophageal cardiac echocardiography for cardioembolic source detection and specialist laboratory tests for rare vascular risk factors. AIS group patients with diagnosed AF and cardioembolic stroke were additionally evaluated using stroke risk assessment scheme CHA_2_DS_2_-VASc [congestive heart failure, hypertension, age ≥ 75 (doubled), diabetes, stroke (doubled), vascular disease, age 65–75, and sex category (female)] [25]. An analysis of all components of CHA_2_DS_2_-VASc score was also performed.

### 2.3. Blood Collection and Laboratory Analysis

For both patients and controls, blood sampling was performed in the morning after an overnight fast. In case of the AIS group, blood samples were collected at the acute phase of stroke (0–24 h). Laboratory tests included complete blood count and serum biochemistry as follows: glucose, total cholesterol, triglycerides, HDL-cholesterol, high sensitive C-reactive protein (CRP), creatinine, fibrinogen, uric acid, alanine aminotransferase (ALAT), and aspartate aminotransferase (ASPAT). All biochemical analyses were performed in Central Hospital Laboratory using an ADVIA 1800 analyser (Siemens), and blood counts were evaluated by means of ADVIA 2120 analyser (Siemens). LDL-cholesterol was calculated using the Friedewald formula for participants with triglycerides < 400 mg/dL.

Additional blood samples were obtained for the measurement of the following adipokines: adiponectin, resistin, and leptin. Blood tubes were centrifuged at 3000*g* for 10 minutes within 30 minutes of collection. Serum samples were immediately separated and stored in a deep freezer at −80°C until use. Total serum adipokine levels were measured using commercially available kits: TECO® Total Human Adiponectin ELISA Kit, MEDIAGNOST Resistin ELISA E50 Kit, and TECO Total Human Leptin ELISA Kit. Absorbance was measured using a spectrophotometer (Bio-Rad) at 450 nm wavelengths.

### 2.4. Statistical Methods

Statistical analyses were performed using SPSS (Statistical Package for Social Sciences, version 20). In the analyses of basic demographic, laboratory, and clinical data, mean values, standard deviations, and percentage values were taken into consideration. Independent sample *t*-test and a two-sample Mann-Whitney *U* test were used for parametric and nonparametric comparisons, respectively. Chi-square test was used for categorical variables with a Yates' or Fisher's correction. Nonparametric model of the analysis of the ANOVA rank Kruskal-Wallis variance with a multiple comparison test was used for the comparison of adiponectin levels in patients with a different stroke etiology. Correlations between waist circumference and adipokine level and CHA_2_DS_2_-VASc and adipokine level variables were analyzed using Rho Spearman correlation coefficient. In all analyses, statistical significance was defined as *p* < 0.5.

## 3. Results

### 3.1. Characteristics of Study and Control Group Features

Table 1 lists the baseline demographic characteristics, a vascular disease risk factor profile, laboratory results, and adipokine serum levels of the 99 AIS patients and 59 controls. The two study groups did not differ for sex, age, mean waist circumference, BMI, or MetS prevalence. Most vascular risk factor profiles and current use of medication known to affect adipokine levels (ACE-I/ATII and statins) were also similar. In the control group, there were fewer past smokers (*p* < 0.01), as well as persons with a history of AF (*p* < 0.001) and arterial hypertension (*p* < 0.05) but there were more cases with a history of diabetes or impaired glucose metabolism (*p* < 0.05). The variables of blood platelet count, hematocrit, total cholesterol, triglycerides, LDL-cholesterol, uric acid, and ALT did not show any significant differences between either of the groups. The AIS group had a significantly higher fasting serum glucose level, blood white cell count, fibrinogen, creatinine, CRP, and ASPAT levels, while the mean level of HDL-cholesterol was significantly lower. Resistin, leptin, and adiponectin levels were comparable between both evaluated groups. Waist circumference positively correlated with leptin levels (*p* < 0.001) and negatively correlated with adiponectin levels (*p* < 0.001) (Table 2).

### 3.2. Adipokines according to MetS Status

To evaluate the expression and clinical significance of adipokines in ischemic stroke patients with MetS, we first examined the serum adipokine levels in AIS patients and controls with regard to the absence or coexistence of MetS. Both subgroups with MetS had comparable values of all evaluated adipokines. In the case of the absence of MetS, only the leptin level was slightly lower in the AIS group than the control one, but the result was borderline significant (Table 3).

Secondly, we evaluated adipokine levels in the group of AIS patients and in a control group with regard to MetS (Table 3). We detected higher resistin levels in the control group with MetS. Leptin levels were statistically significant in AIS and control groups in case of MetS presence.

### 3.3. Adipokines and Stroke Etiology

Because of highest statistical differences in leptin levels in AIS patients, we first analyzed leptin levels in dependence on all main kinds of stroke etiology with the presence or absence of MetS. The highest significant difference of serum leptin concentration was observed in the cardioembolic group. MetS positive patients had more than three times higher leptin level (26. 75 ± 22.48 versus 7.62 ± 7.23, respectively, *p* < 0.001). In the group of patients with small vessel occlusion, MetS presence was also connected with a higher leptin level, but the results were less statistically significant (24.51 ± 13.50 versus 8.51 ± 11.79, resp., *p* < 0.05).

Therefore, we considered all evaluated adipokine and ischemic stroke subtypes in the constellation of cardioembolic and noncardioembolic strokes.  Table 4 shows the results. The significant difference was observed only in the case of leptin levels. The patients with cardioembolic stroke had a higher serum leptin concentration than those with strokes of the remaining kinds of etiology (*p* < 0.05).

### 3.4. Adipokines and MetS and Risk of Cardioembolic Stroke

Among AIS patients, 46 had chronic or paroxysmal AF. Sixteen of them were free of MetS, and the mean value of CHA2DS2-VASc score in this group was 3.69 ± 1.4. Thirty patients were MetS positive and had a significantly higher (*p* < 0.001) mean value of CHA2DS2-VASc score (4.30 ± 1.6).

**Figure 1:**
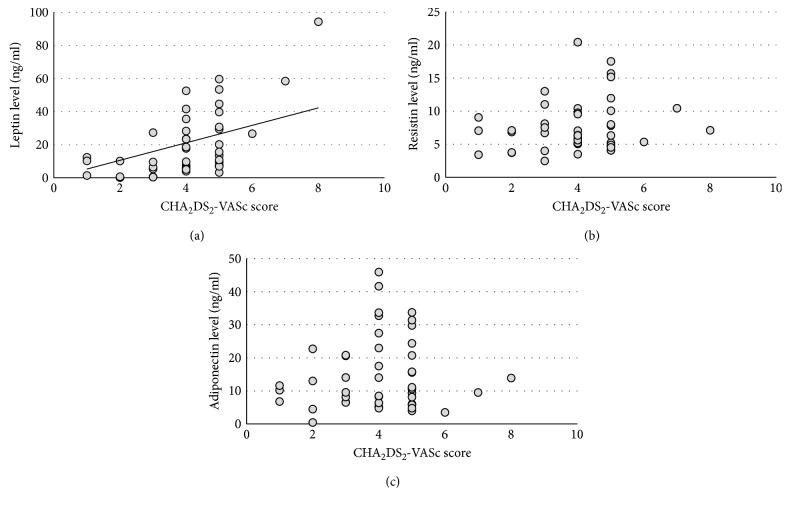
CHA_2_DS_2_-VASc score and serum adipokines concentration.

**Figure 2:**
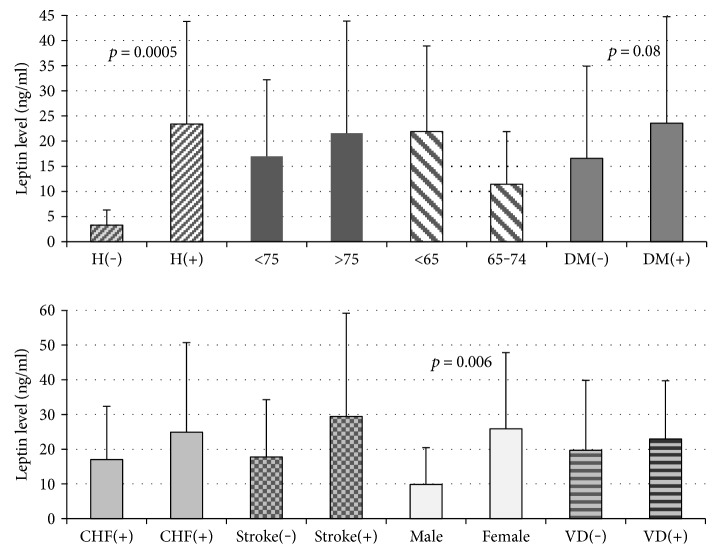
The effect of individual components of CHA2DS2-VASc score on leptin concentration. H: hypertension; <75, >75, <65, and 65–74 refers to patients' age; DM: diabetes mellitus; CHF: congestive heart failure; Stroke: prior stroke or TIA; VD: vascular disease.

A positive correlation between CHA_2_DS_2_-VASc score and serum leptin concentration was found (*p* < 0.001).  Figure 1 shows their linear relationship. In contrast, lack of correlations was seen between CHA_2_DS_2_-VASc score and resistin and adiponectin levels (Table 5). An analysis of individual components of the CHA_2_DS_2_-VASc score showed that hypertension, female gender, and diabetes had the greatest impact on the elevated serum leptin level (Figure 2).

## 4. Discussion

The purpose of this study was to investigate the role of adipokines as novel risk factors for ischemic stroke. Their involvement in the pathogenesis of diabetes, dyslipidemia, inflammation, and coronary artery disease was investigated in several studies, and a number of studies have examined the association of adipokines with the incidence of CHD and combined cardiovascular outcomes [26–28]. However, the results of the adipokines in ischemic stroke studies are conflicting. There is no inconsistent evidence linking circulating adipokines to ischemic stroke. A recently published review paper from 104 articles also did not give a clear conclusion whether adiponectin, leptin, and resistin levels or the single-nucleotide polymorphisms of their encoding genes are independently associated with stroke risk [29]. The source of this discrepancy can result from the fact that ischemic stroke consists of 3 major subtypes with different etiologies: lacunar, atherotrombotic, and cardioembolic. The risk factors for these subtypes may differ, and for this reason, it can be suspected that different adipokines are involved in different stroke subtypes. Thus, it is probable that conflicting results of an adipokine study depend on which group of ischemic stroke patients is evaluated. Kim et al. documented an association between the development of ischemic stroke and a lower level of adiponectin and a higher level of leptin. They also showed that the strength of associations was more intense in LAA strokes [30]. Their research hypothesis was based on adipokines contribution to atherosclerotic “vulnerable plaque,” and from this reason, they divided all stroke patients only into LAA and non-LAA groups.

In our study, ischemic stroke did not affect adipokine levels when all stroke patients were analyzed as one group. We did not find any significant differences in resistin, leptin, and adiponectin levels between AIS and control groups. This result can follow from a specific choice of the control group. We assumed that ischemic stroke, alongside CHD and peripheral vascular disease, is one of the cardiovascular diseases which are influenced by MetS in their progress. Therefore, we decided to compose a control group with a similar number of MetS cases as in the AIS group. In case of the analysis of both groups for the presence or absence of MetS, all evaluated adipokines revealed similar serum levels. The analysis of the AIS group showed that patients with ischemic stroke with accompanying MetS exhibited a much higher serum leptin concentration than MetS negative patients. It is suggested that leptin can be considered a biomarker of ischemic stroke risk in the group of MetS patients. When further examination of the subtypes of ischemic strokes was performed, we found that these higher serum leptin levels are primarily observed in the cardioembolic AIS patients with coexisting MetS. In this context, it may be inferred that ischemic stroke subtypes should be one of the important considerations in studies investigating the utility of stroke biomarkers.

The impact of MetS on cardiovascular disease outcomes is very high and associated with twofold increase in cardiovascular outcomes and one- and fivefold increase in all-cause mortality [31]. Thus, MetS should not be ignored in cardiovascular clinical studies, especially when fat tissue adipokines are investigated. MetS has been also found to be an independent risk factor for AF, even after the adjustment for traditional risk factors for AF [32, 33]. The AF occurrence in these studies gradually and proportionally increased from subjects with no MetS risk factors to patients who fulfilled all 5 MetS criteria. All MetS components are, as a matter of fact, risk factors for AF. Arterial hypertension has been found in 49% to 90% of the patients with AF [34]. The studies showed that hypertension increased the risk of AF almost twofold [35]. It could be explained by left ventricular hypertrophy and stiffness, which in consequence cause electrical, contractile, and structural remodelling [36]. In turn, type 2 diabetes can be associated with diabetic cardiomyopathy. Its pathogenesis originates from myocardial fibrosis, cardiomyocyte hypertrophy, and the alteration of the myocardial microvascular structure. Diabetic cardiomyopathy impairs diastolic function and increases the risk of heart failure which can contribute to the development of AF [37, 38].The relationship between dyslipidemia and AF is not as clear as in the case of other MetS components. It has been proved that reduced levels of HDL-cholesterol are associated with an increased left ventricular mass, cardiac dysfunction, and the development of heart failure, all of which are risk factors for AF. Therefore, abnormal HDL-cholesterol levels may predispose an individual to AF through a structural change in the atrium [32, 33, 39]. The situation with other lipid fractions is more complicated. Some authors showed an inverse relationship between total cholesterol [40], LDL-cholesterol [39], and triglycerides levels [38] with AF. This “dyslipidemia paradox” can result from the fact that dyslipidemic disturbance occurs mainly until the age of 70, whereas the prevalence of AF continuously increases with age. Abdominal obesity, a key component among “MetS components constellation,” is investigated as a key risk factor for cardiovascular diseases. Obesity increases the prevalence of hypertension, ischemic heart disease, congestive heart failure, and cardiac autonomic dysfunction [41, 42]. The relationship between AF and obesity is also well documented [43, 44]. Obesity has been established as an independent risk factor for the genesis of AF [45, 46]. The risk of AF rises about 8% per BMI unit increment [45]. In a meta-analysis performed on the studies of 6 populations, obesity was associated with a 49% increased risk of AF [46]. The manner in which obesity contributes to an increased risk of AF is unclear but many factors are investigated. Primarily, obese people usually exhibit elevated intra-arterial pressure, atrial dilatation, and hypertonic ventricular dysfunction, which can lead to the development of AF. Moreover, obesity and MetS can induce chronic inflammation and oxidative stress, which also play critical roles in the genesis of AF [47, 48]. It has also been proved that epicardial fat is a key element of AF. It can change cardiac electrophysiology by mechanoelectrical feedback and structural remodelling [49]. Epicardial adipose has also been shown to be an important source of inflammatory mediators [45, 50, 51]. Since epicardial adipose tissue in the vicinity of the atrium can directly contact surfaces of the atrium and pulmonary veins, these adipocytes become the closest source of inflammation and initiators of AF.

Leptin, a hormone that is predominantly produced by adipose tissue and one that links visceral obesity with MetS, may be a good risk marker for cardiovascular diseases, including ischemic stroke. The results of the recently published Framingham study can verify this hypothesis. In this study, leptin levels were not directly related to the overall risk of stroke incidence but there was an inverse association with stroke in the top waist-hip ratio quartile [52]. Positive correlation between WC and leptin levels observed in our study supports this observation. Moreover, leptin can also be secreted in the heart by cardiomyocytes and endothelial cells and acts not only in direct interactions but also locally through autocrine mechanisms [45]. Leptin signalling has been shown to contribute to atrial fibrosis and angiotensin II-evoked AF [53]. However, clinical studies present inconsistent results regarding the association of leptin with cardiovascular diseases and indicate that leptin may have either adverse or beneficial effects on the heart and the vascular system [54–56].

Our study complements previous studies in one novel way. To the best of our knowledge, it is the first evaluation of the correlation between CHA_2_DS_2_-VASc score and leptin levels. CHA_2_DS_2_-VASc score risk stratification scheme is the best form of evaluating the real risk of ischemic stroke in nonvalvular AF patients. This method revealed strong evidence for the dependence of FA-related risk of ischemic stroke on leptin levels. Lack of correlation between CHA_2_DS_2_-VASc score and resistin and adiponectin levels supports the significance of the leptin marker among other adipokines. On the other hand, an inverse correlation between CHA_2_DS_2_-VASc score and adiponectin could be expected. This observation, suggesting lower antioxidant and higher inflammatory conditions, was made in the study conducted by Carnevale et al. [57]. CHA_2_DS_2_-VASc score summarizes cardiovascular risks. A statistically significant correlation between CHA_2_DS_2_-VASc score and leptin concentration in cardioembolic stroke patients appears to be consistent with those of CHA_2_DS_2_-VASc score components in which higher leptin levels were found in earlier research such as hypertension, female gender, and diabetes [5, 17, 21, 37, 58]. In addition, our study revealed that lower leptin levels were found only in one higher scoring component, that is, patients > 75 years compared to patients aged 65–74. Arterial hypertension and diabetes are ischemic stroke risk factors included in the so-called “Big Five.” They are also two out of five components of MetS. Leptin, whose serum concentration positively correlates with waist circumference, emphasizes the significance of abdominal obesity in the pathology of arterial hypertension and glucose metabolism disorders. There have also been reports that abdominal obesity is more often present in women [59], and this can be a reason why AF is more often observed in them.

Some potential limitations should be addressed when interpreting the results of this study. First, our sample size was restricted to 99 stroke patients, which was not large enough for comprehensive analysis. Hence, these results should be applied to the general population with caution. Second, although the leptin level is considered to correlate with sex, we did not exclude its effect in each subgroup. Our results should be also interpreted with caution owing to the relatively limited number of cases other than cardioembolic stroke cases. We believe, however, that the great number of cardioembolic strokes results from our good diagnostic procedures with the use of monitoring ECG and reclassification of some patients from the group of cryptogenic to cardioembolic stroke in case FA was found. Specifically designed studies are needed to elucidate the role of leptin in MetS and FA-related ischemic stroke.

## 5. Conclusions

This study documented that leptin could be a potential biomarker for risk stratification of cardioembolic stroke in MetS patients and that the heterogeneity of stroke subtypes should be considered for more refined and precise clinical stroke studies. It also seems that AF and cardioembolic stroke should be included in this group of after effects. Larger prospective studies, both in the general population and in the patients with a history of stroke, are needed to determine whether the measurement of leptin serum levels in case of MetS and FA presence could improve stroke prediction. If this relationship is proven, therapeutic interventions targeting the leptin level might represent a novel approach to reducing cardioembolic stroke incidence. Prevention and treatment of hypertension, dyslipidemia, obesity, and metabolic disorders may play an important role in reducing the risk of AF cardioembolic stroke in the general population. This strategy should be based on healthy lifestyle including weight loss, regular physical activity, and dietary changes and should be supported by appropriate pharmacological intervention. Measurements of serum leptin levels could be a valuable clue helpful in achieving therapeutic goals.

## Figures and Tables

**Table 1:** Baseline demographic, clinical, and laboratory characteristics of stroke cases and matched controls.

	Cases (*n* = 99)	Control (*n* = 59)	*p*
Gender, M/F	47/52	30/29	0.68
Age, mean (SD), y	70.7 (12.6)	67.8 (10.2)	0.06
Arterial hypertension (%)	73 (73.7)	34 (57.6)	<0.05
Diabetes/impaired glucose metabolism (%)	24 (24.2)	23 (39.0)	<0.05
Atrial fibrillation (%)	46 (46.5)	3 (5.1)	<0.001
Ischemic heart disease (%)	28 (28.3)	9 (15.3)	0.06
Congestive heart failure (%)	8 (8.1)	1 (1.7)	0.09
Ischemic stroke/TIA	22 (22.2)	—	—
Hypercholesterolemia (%)	19 (19.2)	11 (18.6)	0.96
Peripheral vascular disease (%)	5 (5.1)	—	—
Current smoker (%)	27 (27.6)	12 (20.7)	0.34
Past smoker (%)	33 (52.4)	15 (26.3)	<0.01
Current drinker (%)	12 (13.0)	3 (5.1)	0.11
Metabolic syndrome (%)	50 (50.5)	30 (50.8)	0.97
ACE-1/AT-II (%)	57 (57.6)	27 (45.8)	0.15
Statins (%)	16 (16.3)	10 (16.9)	0.92
BMI (kg/m^2^)	27.5 (5.3)	26.3 (4.2)	0.18
Waist circumference (cm)	95.2 (15.5)	95.9 (13.2)	0.71
Fasting serum glucose level, mg/dl (SD)	125.1 (34.8)	100.5 (21.7)	<0.001
Blood white cell count, ×10^9^/l (SD)	8.2 (2.6)	6.2 (1.9)	<0.001
Blood platelet count, ×10^9^/l (SD)	23.3 (8.1)	24.3 (6.8)	0.26
Hematocrit (%)	41.52 (7.9)	40.57 (4.0)	0.30
Total cholesterol, mg/dl (SD)	192.1 (49.2)	204.9 (40.4)	0.06
Triglycerides, mg/dl (SD)	124.6 (67.3)	136.5 (72.7)	0.11
LDL-cholesterol, mg/dl (SD)	118.7 (40.1)	125.2 (33.1)	0.16
HDL-cholesterol, mg/dl (SD)	47.2 (16.2)	51.2 (13.5)	<0.05
Creatinine, mg/dl (SD)	0.98 (0.3)	0.8 (0.3)	<0.001
Uric acid, mg/DL (SD)	5.5 (1.5)	5.5 (1.7)	0.53
hs.C-reactive protein, mg/dl (SD)	9.5 (15)	2.8 (3.5)	<0.001
Fibrynogen, g/L	3.7 (1.2)	3.3 (0.7)	<0.05
ALT, U/L (SD)	23.8 (10.4)	26.7 (20.3)	0.89
ASPAT,U/L(SC)	31.9 (16.7)	25.4 (7.6)	<0.01
Adiponectin, *μ*g/mL (SD)	12.8 (10.0)	12.5 (9.7)	0.62
Resistin, *μ*g/mL (SD)	7.0 (3.6)	6.9 (2.9)	0.55
Leptin, *μ*g/mL (SD)	15.9 (16.5)	17.4 (14.6)	0.25

**Table 2:** Correlations between waist circumference and adipokine levels in the AIS group.

Parameters	AIS patients	*N* = 99
	*R*	*p*
Waist circumference & resistin	0.176	0.081
Waist circumference & leptin	0.442	<0.001
Waist circumference & adiponectin	−0.393	<0.001

**Table 3:** Levels of adipokines in AIS and control groups according to MetS status.

	AIS patients	Control	*p*
Resistin, ng/ml (SD)			
MetS (−)	6.99 (4.09)	5.90 (2.22)	0.42
MetS (+)	6.93 (3.19)	7.88 (3.10)	0.09
*p*	0.93	<0.01	
Leptin, ng/ml (SD)			
MetS (−)	8.95 (9.22)	12.85 (10.85)	0.049
MetS (+)	22.71 (16.50)	21.87 (16.50)	0.94
*p*	<0.001	<0.01	
Adiponectin, ng/ml (SD)			
MetS (−)	14.93 (10.95)	13.46 (7.52)	0.99
MetS (+)	10.71 (8.58)	11.64 (11.44)	0.57
*p*	0.1	0.4	

**Table 4:** Levels of adipokines according to the subject stroke etiology groups of AIS patients.

		Stroke etiology	*N* = 99	ng/ml (SD)		*p*
Resistin		Cardioembolic	41	7.83 (4.18)		0.53
	Noncardioembolic	Large artery atherosclerosis, *n* = 11	58	5.25 (2.25)	6.35 (3.10)	
		Small vessel occlusion, *n* = 15		6.17 (1.68)		
		Other determined etiology, *n* = 4		5.99 (4.03)		
		Undetermined etiology, *n* = 17		7.69 (4.41)		
		Unclassified etiology, *n* = 11		5.75 (2.23)		

Leptin		Cardioembolic	41	19.57 (20.53)		<0.05 (0.04)
	Noncardioembolic	Large artery atherosclerosis, *n* = 11	58	8.44 (7.20)	13.17 (12.36)	
		Small vessel occlusion, *n* = 15		17.04 (14.79)		
		Other determined etiology, *n* = 4		8.34 (3.61)		
		Undetermined etiology, *n* = 17		15.10 (14.29)		
		Unclassified etiology, *n* = 11		11.42 (10.73)		

Adiponectin		Cardioembolic	41	14.57 (11.34)		0.14
	Noncardioembolic	Large artery atherosclerosis, *n* = 11	58	8.81 (7.95)	11.55 (8.83)	
		Small vessel occlusion, *n* = 15		15.65 (11.67)		
		Other determined etiology, *n* = 4		5.25 (3.58)		
		Undetermined etiology, *n* = 17		10.34 (6.43)		
		Unclassified etiology, *n* = 11		12.87 (8.18)		

**Table 5:** Correlations between CHA_2_DS_2_-VASc score and levels of adipokines.

Parameters	AIS patients with AF, *N* = 46	
	*R*	*p*
CHA_2_DS_2_-VASc score & resistin	0.215	0.152
CHA_2_DS_2_-VASc score & leptin	0.492	<0.001
CHA_2_DS_2_-VASc score & adiponectin	0.004	0.982
